# Laparoscopic cytoreductive surgery and hyperthermic intraperitoneal chemotherapy for gastric cancer with intraoperative detection of limited peritoneal metastasis: a Phase II study of CLASS-05 trial

**DOI:** 10.1093/gastro/goae001

**Published:** 2024-02-21

**Authors:** Tian Lin, Xinhua Chen, Zhijun Xu, Yanfeng Hu, Hao Liu, Jiang Yu, Guoxin Li

**Affiliations:** Department of General Surgery & Guangdong Provincial Key Laboratory of Precision Medicine for Gastrointestinal Tumor, Nanfang Hospital, The First School of Clinical Medicine, Southern Medical University, Guangzhou, Guangdong, P. R. China; Department of General Surgery & Guangdong Provincial Key Laboratory of Precision Medicine for Gastrointestinal Tumor, Nanfang Hospital, The First School of Clinical Medicine, Southern Medical University, Guangzhou, Guangdong, P. R. China; Department of General Surgery & Guangdong Provincial Key Laboratory of Precision Medicine for Gastrointestinal Tumor, Nanfang Hospital, The First School of Clinical Medicine, Southern Medical University, Guangzhou, Guangdong, P. R. China; Department of General Surgery & Guangdong Provincial Key Laboratory of Precision Medicine for Gastrointestinal Tumor, Nanfang Hospital, The First School of Clinical Medicine, Southern Medical University, Guangzhou, Guangdong, P. R. China; Department of General Surgery & Guangdong Provincial Key Laboratory of Precision Medicine for Gastrointestinal Tumor, Nanfang Hospital, The First School of Clinical Medicine, Southern Medical University, Guangzhou, Guangdong, P. R. China; Department of General Surgery & Guangdong Provincial Key Laboratory of Precision Medicine for Gastrointestinal Tumor, Nanfang Hospital, The First School of Clinical Medicine, Southern Medical University, Guangzhou, Guangdong, P. R. China; Department of General Surgery & Guangdong Provincial Key Laboratory of Precision Medicine for Gastrointestinal Tumor, Nanfang Hospital, The First School of Clinical Medicine, Southern Medical University, Guangzhou, Guangdong, P. R. China

**Keywords:** gastric cancer, peritoneal metastasis, laparoscopic cytoreductive surgery, hyperthermic intraperitoneal chemotherapy

## Abstract

**Background:**

Systemic chemotherapy for gastric cancer with peritoneal metastasis has limited clinical benefit; for those with intraoperative detection of occult peritoneal metastasis, cytoreductive surgery followed by hyperthermic intraperitoneal chemotherapy (HIPEC) is an alternative treatment. However, the feasibility and effects of this modality and criteria for selecting suitable groups remain unclear. This study aimed to explore the safety and efficacy of laparoscopic cytoreductive surgery (L-CRS) followed by HIPEC in gastric cancer with limited peritoneal metastasis, and this study also aimed to determine the optimized cut-off of the peritoneal cancer index.

**Methods:**

Between March 2017 and November 2019, patients diagnosed with gastric cancer peritoneal metastases by using laparoscopy and the Sugarbaker peritoneal cancer index of ≤12 were eligible for inclusion. All patients received L-CRS (including gastrectomy with D2 lymph node dissection) and resection of visible peritoneal metastasis, followed by post-operative HIPEC, and systemic chemotherapy. The primary end points were median progression-free survival and median survival time, and the secondary outcomes were morbidity and mortality within 30 days after surgery.

**Results:**

Thirty patients were eligible for analysis, of whom 19 (63.3%) were female, and the overall mean age was 53.0 years. The post-operative morbidity was 20% and the severe complication rate was 10%. The median survival time was 27.0 months with a 2-year overall survival rate of 52.3% and median progression-free survival was 14.0 months with a 2-year progression-free survival of 30.4%.

**Conclusions:**

L-CRS followed by HIPEC can be safely performed for gastric cancer with limited peritoneal metastasis and potential survival benefits.

## Introduction

Peritoneal metastasis (PM), a hallmark of incurable advanced gastric cancer (GC), currently has no curative therapy. Synchronous PM was found in 5.3%–14.0% of newly diagnosed GC patients worldwide, and even reached 20.0%–30.0% in advanced GC scheduled for curative resection during laparoscopy exploration [1–3]. Systemic palliative chemotherapy is recommended independently for organ or PM; however, the patients with PM had a low response rate because of the barrier between blood and the peritoneum that would prevent drug penetration in the peritoneal layer. Thus, the optimal treatment for patients with PM remains in doubt [4, 5].

The CONVO-GC1 study demonstrated that surgery aiming for an R0 operation after induction chemotherapy can be deemed an appropriate treatment strategy for stage IV GC. This applies not only to technically resectable metastases, but also to marginally resectable and initially unresectable metastases [6]. To minimize the risk of peritoneal disease progression and improve chemotherapy response, surgery first followed by adjuvant chemotherapy was considered another multidisciplinary strategy in gastric cancer peritoneal metastases (GCPM), especially in those initially diagnosed with limited PM [7].

The REGATTA trial failed to show a survival benefit of reduction surgery in the treatment of advanced GC with a single non-curable factor [8]. However, promising results came from later exploration of cytoreductive surgery (CRS) in selected patients in the AIO-FLOT3 trial, although only a small proportion of subjects had GCPM in this study [9]. While CRS was performed in one-quarter of patients with isolated colorectal PM, it was only undertaken in a limited number of patients with GCPM in high-incidence regions, such as Asia and some European countries, due to limited data on the safety and efficacy of this approach [10–12]. Population-based retrospective studies demonstrated that CRS prolonged the overall survival of patients with GCPM, although some patients experienced a high recurrence rate, making CRS a controversial therapy [5, 13, 14].

Preliminary studies have indicated that the oncologic outcomes of CRS are influenced by distinct patterns of peritoneal disease and treatment modality [7], with post-operative hyperthermic intraperitoneal chemotherapy (HIPEC) adding value to the procedure by reducing the risk of recurrence [7, 15]. HIPEC was effective in the eradication of cancer cells for the direct administration of chemotherapeutic drugs into the peritoneal cavity and might be beneficial in selected patients with PM or ascites as demonstrated in the PHOENIX-GC trial [16]. Additionally, the effect of HIPEC in preventing peritoneal dissemination after curative gastrectomy in locally advanced GC has been verified by several retrospective cohorts [11–13] and the ongoing PILGRIM randomized trial [17].

Inspired by recent advances in the treatment modality of GC with low-volume PM, in this prospective study, we aimed to explore the safety and efficacy of CRS followed by HIPEC and systemic chemotherapy in GC patients with intraoperative detection of limited PM. Here, we report the results of the Phase II CLASS-05 trial.

## Patients and methods

### Study design

The CLASS-05 trial was an investigator-initiated, Phase II/III, randomized–controlled trial (NCT03023436). The trial protocol was approved by the institutional review board of Nanfang Hospital, Southern Medical University, Guangzhou, China. All the participants provided written informed consent. Eligible participants were 18–75 years old, had histologically proven primary gastric adenocarcinoma with intraoperative detection of PM, and were evaluated by using laparoscopic exploration. HER2-neu overexpressing adenocarcinoma was excluded for confirmed effects of trastuzumab in this group of patients. Detailed inclusion and exclusion criteria are shown in Supplementary Table S1.

### Study procedures

#### Evaluation and enrollment

Patients diagnosed with locally advanced GC (cT3-cT4a) at preoperative evaluation, including an adrenal computed tomography scan, and without evidence of non-curable tumor factors were potential subjects. Peritoneal disease was evaluated according to the Sugarbaker peritoneal cancer index (PCI) score, including the number, size, and region of the tumor, which were determined by diagnostic laparoscopy [18]. Patients with a PCI score of ≤12 and expected CRS to achieve no macroscopic residual tumor (completeness of cytoreduction score, CC0) were enrolled intraoperatively. The location of the PM was classified according to the Japanese Classification of Gastric Carcinoma (JCGC) 15th edition [19].

#### Surgical quality control

Experienced surgeons who have performed >300 cases of laparoscopic gastrectomy for patients with advanced GC participated in this trial. Surgical quality control was maintained by using mandatory intraoperative video and assessed by the investigators. In total, laparoscopic or laparoscopic-assisted surgery were both acceptable in this trial and the extent of the gastrectomy and D2 lymph node dissection were performed according to the Japanese Gastric Cancer Treatment Guidelines (5th edition) [20].

#### Surgical procedures and chemotherapy

L-CRS with curative intent includes a gastrectomy with D2 lymph node dissection and en-bloc resection of all visible peritoneal tumors [21–23]. Post-operative HIPEC was performed using the closed abdomen technique according to the Chinese expert consensus [24] and docetaxel (75 mg/m^2^) was applied [25]. Patients received the first cycle of systemic chemotherapy within 6 weeks after surgery and a chemotherapy regimen of platinum drugs combined with a fluoropyrimidine was recommended. Maintenance chemotherapy or follow-up after stable disease status and second-line systemic therapy after disease progression were both determined by physicians.

### Study end point

This trial evaluated the safety of L-CRS followed by HIPEC in patients with GC and intraoperative detection of PM. The primary outcome was median survival time. Secondary outcomes included the 2-year overall survival (OS) rate and the progression-free survival (PFS) rate, morbidity and mortality within 30 days after surgery. Here, we report the preliminary results of morbidity and mortality, and short-term outcomes. Complications were diagnosed by using imaging examination or obvious clinical evidence as described before, and the severity of the post-operative complications was assessed according to the Clavien–Dindo classification [26, 27]. Currently, we report the results of the Phase II trial.

### Statistical analysis

To evaluate the safety, technical, and procedural success of the combination of L-CRS and post-operative HIPEC and chemotherapy in GCPM patients, the sample size of the CLASS-05 Phase II trial was set as 30 cases according to the IDEAL framework [28]. Statistical analysis was conducted using the SPSS 19 software package. The analysis focused on patients enrolled who received L-CRS followed by at least one cycle of HIPEC. Descriptive statistics were used and the data are presented as the mean with standard deviation (SD) or median with range for continuous variables, and as numbers followed by percentages for categorical variables. OS and PFS were estimated by using Kaplan–Meier curves and a Cox regression model was used to estimate the hazard ratio (HR) and two-sided 95% confidence intervals (CIs). The optimum cut-off score for PCI was determined on the basis of the association with the patients’ OS by using X-tile as described previously [29]. A two-sided *P *≤ 0.05 was considered statistically significant.

## Results

### Patient characteristics

Between 8 March 2017 and 27 November 2019, 34 patients diagnosed with GCPM intraoperatively were enrolled. After excluding 4 patients who did not receive any intraperitoneal chemotherapy, 30 patients remained in the as-treated population. The complete clinical and pathological characteristics of the patients are shown in Supplementary Table S2. The mean age (SD) was 53.0 (11.7) years and the mean body mass index (SD) was 22.3 (3.5) kg/m^2^. All of patients enrolled in this trial had an Eastern Cooperative Oncology Group performance status of 0 or 1.

### Surgery and HIPEC

All of the enrolled patients received laparoscopic distal (15 of 30, 50.0%) or total (15 of 30, 50.0%) gastrectomy (Table 1). D2 lymph node dissection, regional treatment of peritoneal metastatic sites, and CC0 resection were achieved. Combined resection was performed in two patients, including one case of GC invading the partial transverse colon and one case diagnosed with a benign ovarian cyst. The average operation time (SD) was 253.6 (49.6) min; no intraoperative complication was observed. Most patients (25 of 30, 83.3%) received two or more cycles of HIPEC after surgery.

**Table 1.** Pathologic characteristics and surgical outcomes of 30 patients with gastric cancer

Characteristic	Value
Location of primary tumor, *n* (%)	
Lower third	18 (60.0)
Middle third	7 (23.3)
Upper third	5 (16.7)
Histology, *n* (%)	
Signet-ring cell	13 (43.3)
Others	17 (56.7)
Pathological T stage, *n* (%)	
T3	2 (6.7)
T4a	28 (93.3)
Number of retrieved lymph nodes, median (range)	51.5 (24–90)
Gastrectomy, *n* (%)	
Distal	15 (50.0)
Total	15 (50.0)
Additional organ resection, *n* (%)	
None	28 (93.3)
Partial transverse colon, *n* (%)	1 (3.3)
Ovary	1 (3.3)
Residual tumor, *n* (%)	
R0	28 (93.3)
R1	2 (6.7)
Location of metastasis (P stage), *n* (%)	
P1a	13 (43.3)
P1b	10 (33.3)
P1c	7 (23.3)
Peritoneal carcinomatosis index, *n* (%)	
1–3	17 (56.7)
4–6	7 (23.3)
7–12	6 (20.0)
Time to first ambulation, days, median (range)	2 (1–2)
Time to first flatus, days, median (range)	3 (2–4)
Time to first liquid intake, days, median (range)	4 (3–4)
Time of post-operative hospital stay, days, median (range)	8 (8–10)

### PM status and pathological characteristics

With laparoscopy exploration, patients were classified into three groups: P1a (13 of 30, 43.3%), P1b (10 of 30, 33.3%), and P1c (7 of 30, 23.3%). Among the 30 patients, 24 (80.0%) were evaluated as having regional PM with a PCI of ≤6, whereas 6 patients (20.0%) were evaluated as having a moderate extent of metastasis (PCI > 6; Table 1). The mean PCI score (SD) was 2.7 (1.1) in P1a, 4.1 (2.3) in P1b, and 8.0 (2.1) in P1c patients, with a significant difference between the P1a or P1b and P1c populations (*P *<* *0.001).

Gastrectomy with a negative resection margin was achieved in 28 patients (93.3%); 2 patients (6.7%) received R1 resection with a positive proximal margin. Pathologic diagnosis confirmed T4b invasion tumor in 43.3% (13/30) of patients with invasive structures such as the pancreas capsule, anterior lobe of the transverse mesocolon, diaphragm, and hepatoduodenal ligament. The median number of retrieved lymph nodes (range) was 51.5 (24–90) and the median positive number (range) was 10.0 (0–72), with 21 patients (70.0%) diagnosed with N3 stage (Table 1).

### Post-operative recovery and complications

The median time to first ambulation (range) was 2 (1–2) days, to first flatus was 3 (2–4) days, to first liquid intake was 4 (3–4) days, and the post-operative hospital time was 8 (8–10) days. Post-operative complications were observed in six patients (20.0%), including ileus, transverse colon leakage, intra-abdominal bleeding, and pleural effusion. According to the Clavien–Dindo classification, three cases (10.0%) were classified as stage II, two cases (6.7%) were classified as stage IIIa, and one case (3.3%) was classified as stage IIIb.

### Chemotherapy and adverse effects

Characteristics of first-line chemotherapy are shown in Table 2; 26 patients (86.7%) received post-operative chemotherapy. The average time to commencing chemotherapy after surgery (SD) was 34.4 (8.9) days. Delayed initiation of chemotherapy (chemotherapy was not administered until 6 weeks after the surgery) was observed in four patients, two of whom experienced Grade III post-operative complications. Table 2 displays the number of chemotherapy cycles administered. Three patients experienced Grade 3 or higher adverse events during first-line therapy, while four patients encountered Grade 1 or 2 adverse events.

**Table 2.** Morbidity and mortality of 30 patients with gastric cancer after operative treatment

Characteristic	Value
Post-operative complication, *n* (%)	
None	24 (80.0)
One or more	6 (20.0)
Anastomotic leakage, *n* (%)	1 (3.3)
Intra-abdominal bleeding, *n* (%)	2 (6.7)
Ileus, *n* (%)	2 (6.7)
Pulmonary infection, *n* (%)	1 (3.3)
Clavien–Dindo classification, *n* (%)	
II	3 (10.0)
IIIa	2 (6.7)
IIIb	1 (3.3)
HIPEC cycles, *n* (%)	
1–2	16 (53.3)
3–4	14 (46.7)
First-line chemotherapy cycles, *n* (%)	
0–3	11 (36.7)
4–6	7 (23.3)
>6	12 (40.0)
Chemo-related adverse events, *n* (%)	
None	23 (76.7)
Grade 1/2	4 (13.3)
Grade 3/4	3 (10.0)
Recurrence, *n* (%)	
Multiple sites	8 (26.7)
Uncertain	7 (23.3)
Local	1 (3.3)
Liver	2 (6.7)
Ovary	1 (3.3)

HIPEC = hyperthermic intraperitoneal chemotherapy.

### Survival outcomes

**Figure 1.**
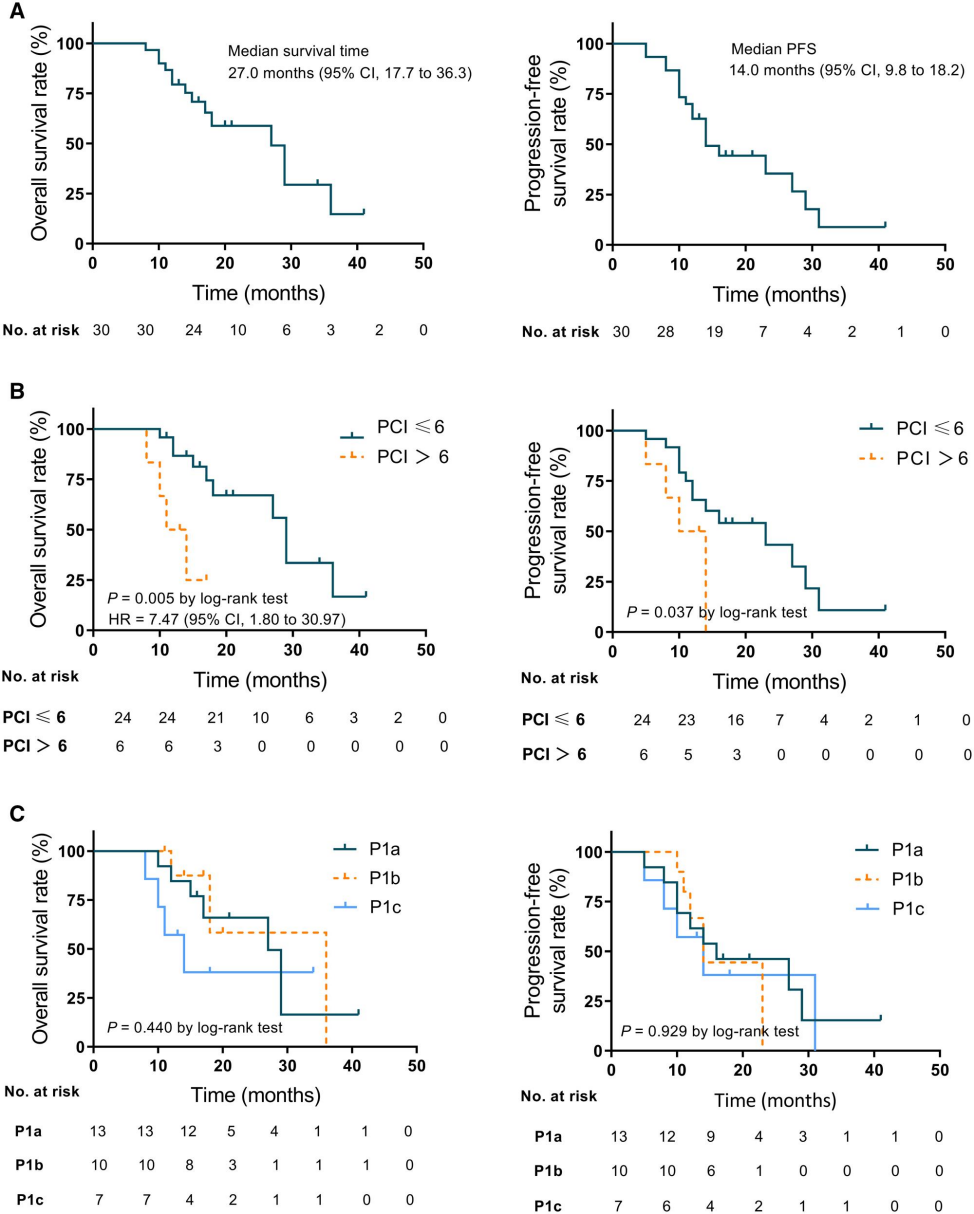
Overall survival (OS) and progression-free survival (PFS) in all patients. (A) Kaplan–Meier analysis of OS and PFS in all patients. (B) Differences in OS and PFS between patients with PCI of ≤6 and >6. (C) Differences in OS and PFS between patients with P1a, P1b, and P1c peritoneal metastasis.

After a median follow-up time of 20.0 months (range, 8.0–41.0 months), 15 patients died; the median survival time of 30 patients in this study was 27.0 months, with 1- and 2-year survival rates of 79.4% and 52.3%, respectively (Figure 1A). Recurrence was recorded in 12 patients; the most frequent event was multiple-site recurrence, followed by local, liver, and ovary recurrence (Table 2). The number of recurrences or cancer-related deaths was 19; the median PFS was 14.0 months, with 1- and 2-year survival rates of 62.6% and 30.4%, respectively (Figure 1A).

In the analysis of predictors of OS, we performed X-tile analysis and classified patients into high-PCI and low-PCI groups with a PCI of 6 as the optimized cut-off (data not shown). To assess the distribution of PCI grades in the survival of GCPM, univariate analysis was performed, and it showed that a high PCI grade indicated poor survival with gastrectomy range and HIPEC cycles. Multivariate Cox regression analysis identified high PCI grade (HR = 7.5, 95% CI 1.8–31.0) and total gastrectomy (HR = 4.0, 95% CI 1.2–13.2) as independent prognostic factors for poor survival (Table 3). The median survival times for the low-PCI and high-PCI groups were 29.0 months (95% CI 26.4–31.6 months) and 11.0 months (95% CI 7.2–14.8 months, log-rank *P *=* *0.005, Figure 1B), respectively, and the median PFS times for the two groups were 23.0 months (95% CI 9.7–36.7 months) and 10.0 months (95% CI 6.4–13.6 months, log-rank *P *=* *0.037), respectively. There was no significant difference in survival or PFS between the different P-stage groups (Figure 1C).

**Table 3.** Cox regression analysis of overall survival

		Univariate		Multivariate	
Variable	Category	HR (95% CI)	*P*-value	HR (95% CI)	*P*-value
Location of primary tumor	Lower third	Reference			
	Middle third	1.27 (0.36–4.44)	0.711		
	Upper third	0.97 (0.22–4.21)	0.964		
Location of metastasis (P stage)	P1a	Reference			
	P1b	0.74 (0.19–2.91)	0.666		
	P1c	1.83 (0.52–6.36)	0.345		
Pathological T stage	T4a vs T3	31.27 (0.03–29,913.45)	0.326		
Pathological N stage	N3 vs N0–2	1.55 (0.42–5.68)	0.506		
Surgical radicalness	R1 vs R0	3.13 (0.36–27.06)	0.299		
Gastrectomy range	Total vs distal	3.21 (1.00–10.30)	0.050	3.95 (1.18–13.18)	0.026
Peritoneal carcinomatosis index	>6 vs ≤6	5.56 (1.45–21.27)	0.012	7.47 (1.80–30.97)	0.005
HIPEC cycles	3–4 vs 1–2	3.95 (1.10–14.21)	0.036	Not in equation	
Post-operative complication	Yes vs No	1.09 (0.30–4.05)	0.892		
Chemotherapy cycles	>6 vs ≤6	0.87 (0.29–2.61)	0.808		
Chemotherapy regimen	Capecitabine or uncertain	Reference			
	CapeOX	0.97 (0.19–5.03)	0.967		
	DOF or DOC	1.28 (0.24–6.74)	0.773		

HR = hazard ratio, CI = confidence interval, HIPEC = hyperthermic intraperitoneal chemotherapy, DOF = docetaxel, oxaliplatin, 5-fluorouracil, DOC = docetaxel, oxaliplatin, capecitabine, CapeOX = capecitabine and oxaliplatin.

## Discussion

In this pilot study, CRS followed by post-operative HIPEC and chemotherapy yielded a considerable median OS of 27.0 months and median PFS of 14.0 months in patients with limited PM. Although we could not directly compare our findings with those of other reports, improved survival time seems to be associated with this modality. More importantly, CRS followed by HIPEC was demonstrated to be safe and feasible with an acceptable rate of complications, which will encourage future randomized–controlled trials (RCTs) to explore its value in selected patients.

There has been increasing evidence supporting the important role of CRS and HIPEC in the treatment of GCPM. However, the question of whether CRS followed by post-operative HIPEC in a short interval time results in treatment-related morbidity or mortality remains controversial [30]. In the present prospective study, the severe complication rate was 10%, which is comparable to reported data. The most common complications were ileus and intra-abdominal bleeding, which is consistent with the results of retrospective analyses. Recent studies have also explored the toxicity of different drugs applied in HIPEC and hematological toxicity was found to be uncommon [25, 31]. However, the PERISCOPE I study in the Netherlands showed that dose-limiting toxicities of docetaxel were associated with post-operative ileus [15] whereas, in our study, we did not find a significant association between HIPEC treatment and post-operative complications or delayed initiation of systemic chemotherapy. Several ongoing RCTs are evaluating post-operative HIPEC in patients with advanced GC (PILGRIM, GASTRICHIP) [32] or colon cancer (COLOPEC, PRODIGE7) [33]. Preliminary results of these RCTs and the results of our study showed that intraoperative or post-operative HIPEC with oxaliplatin or docetaxel was well tolerated at an appropriate dose [17, 34]. Despite the small sample size and non-RCT design, the present prospective study provided evidence on the safety and feasibility of CRS followed by HIPEC in GCPM patients; however, the maximum tolerated dose and combination of drugs warrant more experimental and clinical studies to determine the most efficient regimen.

CRS followed by HIPEC was developed as an ideal treatment strategy in peritoneal tumors and is still used worldwide in GCPM after the REGATTA trial [8], which could not show survival benefit of reduction gastrectomy in GC with non-curable factors. The results of the REGATTA trial cannot be extrapolated to CRS as a curative surgery and to the application of HIPEC as adjuvant therapy. We took advantage of both CRS and HIPEC in this study, and found that survival benefit was linked to surgery with curative intent, immediate application of adjuvant HIPEC, and strict selection of patients.

Due to the lack of high-level evidence, the major skepticism is about the value of surgery in GCPM and the goal of the surgery [35]. In our study, all patients received D2 resection and macroscopic complete resection of PM. Prolonged survival was observed, especially in those who were more likely to undergo curative surgery (PCI ≤ 6). Recently, the AIO-FLOT3 trial proposed that the goal of surgery (curative or palliative) was strongly linked to the survival benefit of surgery [9]. In the AIO-FLOT3 study [9], retroperitoneal lymph node metastases and liver metastases constituted the two largest subgroups, and only patients with adequate response to preoperative chemotherapy underwent surgery, which was associated with a realistic chance of curative surgery [17, 34]. The necessity for receiving CRS for patients with GCPM was confirmed in a large retrospective cohort study CYTO-CHIP and survival improvement was observed in patients receiving CC-0 or CC-1 CRS [13]. Thus, our prospective study provides more instructive evidence for beneficial strategies based on CRS in GCPM.

Additionally, HIPEC was performed in this study to reduce peritoneal recurrence. Even complete macroscopic CRS is insufficient to improve the long-term survival of GCPM, and HIPEC is already being used outside the trial [36–39]. However, various conclusions have been reported in the existing research. The PHOENIX-GC trial did not show statistical superiority of intraperitoneal plus systemic chemotherapy in GCPM [16]. However, it was supposed that the extent of PM and the absence of surgical intervention might offset the survival benefit of intraperitoneal chemotherapy [16]. We prospectively verified an absolute requirement for HIPEC after CRS in improving patient survival and preventing peritoneal recurrence, which is consistent with the results reported in the CYTO-CHIP study [13]. Additionally, considering that a single exposure of intraperitoneal chemotherapy is too short to obtain clinical effect, post-operative but not intraoperative HIPEC was delivered and most patients received more than one cycle of HIPEC, with improved PFS and a lower surgical complication rate. Currently, there is only one completed RCT evaluating the efficacy of HIPEC after CRS, but the survival rate was modest and patients with severe peritoneal disease were enrolled in this small sample trial [40]. The German GASTRIPEC trial was terminated due to difficulty in enrollment [41], but PERISCOPE II will answer the most important question in comparing HIPEC with standard systemic chemotherapy [42].

In addition to a well-designed treatment strategy, the extent of PM is a decisive factor for favorable prognosis, which is closely linked to the completeness of CRS and the risk of recurrence. The optimized PCI cut-off level indicating potential survival benefit was reported to be <6, <12 or <20 in patients receiving CRS and HIPEC [43]. In a meta-analysis including nine trials evaluating the efficacy of complete and incomplete CRS in GCPM, Coccolini *et al*. [44] proposed a hypothetical PCI cut-off value of 12 for the intent of obtaining CC0 surgery and better survival. In our prospective study, the laparoscopic PCI stage was administered to provide a measurable burden of peritoneal lesions, and L-CRS was successfully performed in patients with PCI limited to 12. As a result, 93% of them received CC0 resection, which was similar to reported data with an acceptable morbidity rate. However, we supposed that the PCI should be limited to 6 for a survival benefit according to the subsequent cut-off point optimization and stratification analysis. The different cut-off values revealed the dissociation of completeness of CC-0 resection and survival benefit, which could partly explain why different cut-off values were recommended by different studies [44]. Additionally, it is difficult to compare the results because the PCI is a quantitative score. A highlight of this study is that a stricter limitation of PCI was verified to be essential to achieve oncological benefit by using L-CRS compared with that for complete stripping of the peritoneum by using open surgery. We also discovered that L-CRS is feasible for patients with a limited extent of GCPM, offering an enlarged visible area and flexible operation in the cavity, which results in a minimally invasive procedure and faster recovery time compared with open surgery. Furthermore, potential improvements in post-operative HIPEC and chemotherapy compliance can also be expected.

There were several limitations in our study. First, we did not design a dose-escalation study to determine the most effective regimen and tolerated dose of HIPEC in GCPM patients because docetaxel is widely approved in intraperitoneal chemotherapy in China. Second, to decrease the difficulties in enrollment, we did not limit the regimens of systemic chemotherapy, which was indispensable for systemic control of the disease. This study showed the benefit of immediate HIPEC after surgery in GC, but did not show whether systemic chemotherapy or local treatment by using CRS should be performed first. Third, due to the lack of active surveillance by using second-look laparoscopy, a delayed diagnosis of progression of disease progression existed. Additionally, the optimized cut-off value of PCI was determined based on a small sample design and further verification in a larger population is warranted.

Taken together, this Phase II pilot study showed that L-CRS followed by HIPEC in patients with low-volume PM can be safely performed with potential survival benefits. The following Phase III multicenter RCT would provide more reliable evidence regarding the efficacy of this modality.

## Supplementary Material

goae001_Supplementary_Data
