# Supplementary material for: Laparoscopic cytoreductive surgery and hyperthermic intraperitoneal chemotherapy for gastric cancer with intraoperative detection of limited peritoneal metastasis: a Phase II study of CLASS-05 trial
Source: Gastroenterol Rep (Oxf). 2024 Feb 21;12:goae001. doi: 10.1093/gastro/goae001 (PMC10882263; doi:10.1093/gastro/goae001)
Supplement: goae001_Supplementary_Data [file goae001_supplementary_data.zip › 2023-206_Supplementary_Table_S1.doc]

**Supplementary Table S1. Inclusion and Exclusion criteria**

| Inclusion criteria | Exclusion criteria |
| --- | --- |
| - 18 years < age < 75 years | - Adenocarcinoma of esophageal-gastric junction (AEG) that requires thoracotomy |
| - Histologically proven primary gastric adenocarcinoma | - Distant metastases not confined to peritoneum, including liver (H1), para-aortic lymph node (stations 16a1 and/or b2), lung, brain, bone and other organs |
| - Pathological gastric cancer by endoscopic biopsy | - Histologically proven HER2-neu overexpressing adenocarcinoma |
| - Clinical T3-4NxM0 or T3-4NxM1 (M1 limited to peritoneum, P1) at preoperative evaluation according to the AJCC Cancer Staging Manual Seventh Edition | - History of previous neoadjuvant chemotherapy, radiotherapy or clinical trial treatment within 3 months |
| - Primary gastric adenocarcinoma with distant metastases confined to peritoneum (P1) confirmed by laparoscopic exploration and all disease should be deemed resectable and peritoneal cancer index (PCI) is less than 12 | - Cerebrovascular accident occurred within 6 months (myocardial infarction, unstable angina, cerebral infarction, or cerebral hemorrhage) - Contraindication for chemotherapeutic agents or HIPEC |
| - Performance status of 0 or 1 on ECOG (Eastern Cooperative Oncology Group) scale | - Women of child-bearing potential who are pregnant or breastfeeding |
| - Written informed consent | - History of prior/other malignancies within the 5 years prior to enrollment |
|  | - History of continuous systematic administration of corticosteroids within one month - Requirement of simultaneous surgery for other disease |
|  | - Emergency surgery due to complication (bleeding, obstruction or perforation) caused by gastric cancer |
|  | - FEV1 <50% of predicted values |
