# Supplementary material for: Laparoscopic cytoreductive surgery and hyperthermic intraperitoneal chemotherapy for gastric cancer with intraoperative detection of limited peritoneal metastasis: a Phase II study of CLASS-05 trial
Source: Gastroenterol Rep (Oxf). 2024 Feb 21;12:goae001. doi: 10.1093/gastro/goae001 (PMC10882263; doi:10.1093/gastro/goae001)
Supplement: goae001_Supplementary_Data [file goae001_supplementary_data.zip › 2023-206_Supplementary_Table_S2.doc]

^Supplementary Table S2. Clinicopathologic characteristics and surgical outcomes of 30 patients with gastric cancer^

| Characteristic | Value |
| --- | --- |
| Sex, *n* (%) |  |
| Male | 11 (36.7) |
| Female | 19 (63.3) |
| Age, years, mean (SD) | 53.0 (11.7) |
| BMI, kg/m^2^, mean (SD) | 22.3 (3.5) |
| ECOG PS, *n* (%) |  |
| 0 | 26 (86.7) |
| 1 | 4 (13.3) |
| Comorbidity, *n* (%) |  |
| None | 7 (23.3) |
| One or more | 23 (76.7) |
| Symptoms, *n* (%) |  |
| None | 10 (33.3) |
| One or more | 20 (66.7) |
| Tumor size, cm, mean (SD) | 4.4 (1.8) |
| Pathological N stage, *n* (%) |  |
| N0 | 3 (10.0) |
| N1 | 3 (10.0) |
| N2 | 3 (10.0) |
| N3 | 21 (70.0) |
| Metastatic lymph nodes, *n* (%) | 15.8 (18.0) |
| Cytology, *n* (%) |  |
| CY negative | 7 (23.3) |
| CY positive | 2 (6.7) |
| CY unknown | 21 (70.0) |
| Reconstruction approach, *n* (%) |  |
| Billroth-II | 11 (36.7) |
| Roux-en-Y | 19 (63.3) |
| Abbreviations: SD, standard deviation; BMI, body mass index; ECOG PS, Eastern Cooperative Oncology Group Performance Status Scale. | |
